# The role of ultra-high field MRI and image processing in the presurgical workup in MRI-negative focal epilepsy: A validated 7T MRI case study

**DOI:** 10.1016/j.ebr.2025.100761

**Published:** 2025-03-15

**Authors:** Daniel Uher, Gerhard S. Drenthen, Christianne M. Hoeberigs, Rick H.G.J. van Lanen, Albert J. Colon, Roy A.M. Haast, Vivianne H.J.M. van Kranen-Mastenbroek, Guido Widman, Paul A.M. Hofman, Louis G. Wagner, Jan C. Beckervordersandforth, Jacobus F.A. Jansen, Olaf E.M.G. Schijns, Walter H. Backes, on behalf of the ACE study group

**Affiliations:** a Department of Radiology and Nuclear Medicine, Maastricht University Medical Centre, Maastricht, the Netherlands; b Mental Health and Neuroscience Research Institute (MHeNs), Maastricht University, Maastricht, the Netherlands; c Department of Neurosurgery, Maastricht University Medical Center, Maastricht, the Netherlands; d Academic Center for Epileptology, Kempenhaeghe and Maastricht University Medical Centre, Heeze, Maastricht, the Netherlands; e Centre d‘ Etudes et Traitement de l‘Epilepsie, CHU-Martinique, Fort-de-France, France; f Aix-Marseille Université, CRMBM, CNRS UMR 7339, Marseille, France; g Department of Clinical Neurophysiology, Maastricht University Medical Center, Maastricht, the Netherlands; h Department of Epileptology and Neurology, RWTH Aachen University Hospital, Aachen, Germany; i Care and Public Health Research Institute (CAPHRI), Maastricht University, Maastricht, the Netherlands; j Department of Radiology, Horatio Oduber Hospital, Oranjestad, Aruba; k Department of Pathology, Maastricht University Medical Center, Maastricht, the Netherlands; l Department of Electrical Engineering, Eindhoven University of Technology, Eindhoven, the Netherlands

**Keywords:** 7T MRI, Focal cortical dysplasia, Resting-state fMRI, Voxel-based morphometry, ReHo, fALFF, MAP18

## Abstract

This case study demonstrates the value of combined 7 T structural and functional MRI in the presurgical workup of a 24-year-old male with drug-resistant focal epilepsy who was initially considered MRI-negative on clinical 3 T MRI. The patient underwent extensive presurgical workup with 7 T MRI, magnetoencephalography, stereo-electroencephalography, and resection of the suspected right frontal epileptogenic zone. Histopathology showed focal cortical dysplasia (FCD) type IIb. The patient remained 11 months after surgery seizure-free. Retrospective analysis revealed that both structural and functional 7 T MRI showed abnormalities within the resected area. Morphometric Analysis Program (MAP18) detected abnormalities on both 3 T and 7 T images. However, abnormalities were more conspicuous on 7 T. Resting-state functional MRI metrics, particularly regional homogeneity and fractional amplitude of low-frequency fluctuations, demonstrated significantly increased values in both a MAP18-defined region of interest and the entire resected area compared to a healthy control group (p < 0.05). However, extensive unspecific abnormalities were also observed outside the resected region, highlighting the importance of a multimodal approach. This case study illustrates that advanced image processing of ultra-high field structural and resting-state functional MRI scans may enhance the detection of subtle epileptogenic lesions in presurgical evaluation, potentially improving post-operative seizure outcome and associated quality of life.

## Highlights

• Seizure freedom was achieved via resective surgery in an MRI-negative patient.
• Post-operative histopathology confirmed focal cortical dysplasia (FCD) type IIb.
• Retrospective MAP18 analysis showed higher conspicuity at 7 T than 3 T MRI.
• Resting-state fMRI metrics were abnormal in the MAP18-defined FCD region.

## 1 Introduction

Despite advances in imaging technology, up to 30 % of patients with drug-resistant focal epilepsy remain MRI-negative, presenting a significant diagnostic and therapeutic challenge [1], [2]. Epilepsy surgery, with the aim of resecting the epileptogenic zone (EZ), is a potentially curative and evidence-based treatment option [3]. Identification of a lesion on MRI is a major predictive factor for good treatment outcome, i.e. seizure freedom or significant seizure reduction [4].

Focal cortical dysplasias (FCDs) are congenital lesions classified into multiple subgroups by the International League Against Epilepsy (ILAE) [5]. FCDs can be very subtle, and thus difficult to detect in MR images even by specialists [6]. To improve the detection of subtle FCDs, automated morphometry analysis can be utilized, most commonly via the Morphometric Analysis Program (MAP18) which provides an automated analysis of mainly T1-weighted images [7].

Furthermore, ultra-high field (UHF) MRI (>3T) holds a significant promise for better identification and delineation of the lesion due to its better spatial resolution and tissue contrast [8]. Studies have shown that 7 T MRI has an added value in the presurgical workup of MRI-negative patients [9]. Apart from improved structural imaging, it allows for stronger blood-oxygen-level-dependent (BOLD) signal of resting-state functional MRI (rs-fMRI) which serves as a measure of the neuronal activity [10].

This case study highlights the integration of structural and functional 7 T MRI, along with image processing (MAP18 and rs-fMRI metric evaluation), into the presurgical workup may enhance lesion detection, delineation and improve surgical seizure outcome and quality of life in patients with MRI-negative focal epilepsy.

## 2 Materials and methods

Throughout the manuscript, the term “pre-processing” refers to the image processing steps taken prior to the application of MAP18 and rs-fMRI analysis, while the term “post-processing” refers to the MAP18 and rs-fMRI analysis.

### 2.1 Case description

**Fig. 1:**
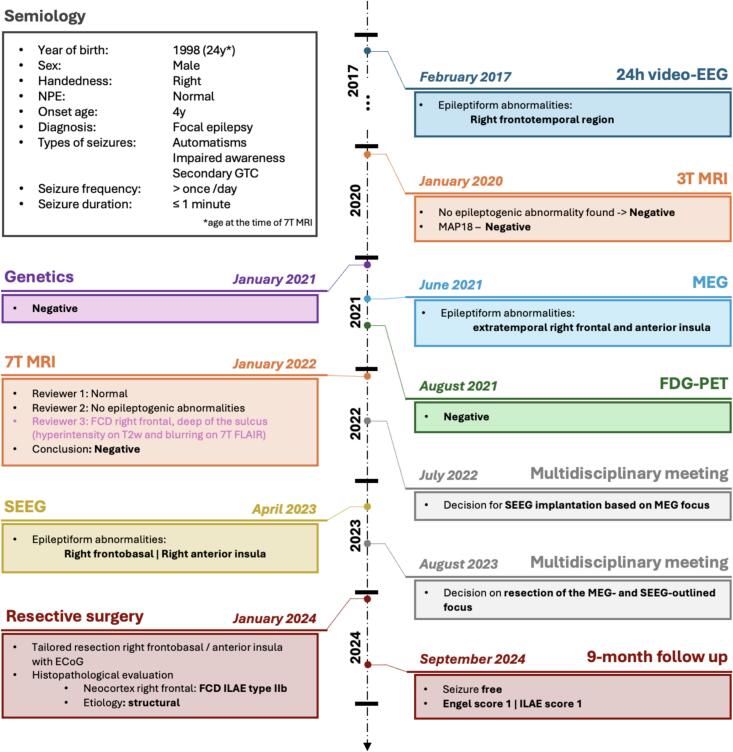
Visualization of the patient’s presurgical trajectory. NPE: neuropsychological evaluation; GTC: generalized tonic-clonic seizures; EEG: electroencephalography; FDG-PET: fluorodeoxyglucose positron emission tomography; MEG: magnetoencephalography; SEEG: stereo-EEG; ECoG: electrocorticography; FCD: focal cortical dysplasia; ILAE: International League Against Epilepsy.

A 24-year-old male with 3 T-MRI-negative focal DRE was referred to the Academic Center for Epileptology Kempenhaeghe-Maastricht UMC + for work-up in the trajectory for epilepsy surgery. The patient’s presurgical workup included a video-EEG, 3 T MRI, genetic screening, positron emission tomography, magnetoencephalography (MEG), and implantation of intracerebral depth electrodes (SEEG). The patient was included into the EpiUltra study [11] and scanned at 7 T in January 2022 according to the study protocol. The study was approved by the local medical ethical committee (trial no. NL66929.068.18; METC18-020) and the patient signed an informed consent prior to scanning and a separate consent form for the publication of this research. The detailed timeline of the workup can be found in Fig. 1.

The neuropsychological evaluation (NPE) in patients selected for epilepsy surgery in our center is scheduled a number of months prior to surgery and 1–2 years after surgery. Both pre- and postoperative evaluations consist of intelligence profile assessment, language and memory functioning, executive function assessment, visual perception, visuo-constructive skills, and self-reported quality of life questionnaires.

### 2.2 Scanning protocol

The patient received a 3 T MRI (Philips Achieva dStream, The Netherlands) using a 32-channel receive phase-array head coil. The acquired sequences were T1-weighted (T1w), T2-weighted, and fluid-attenuated inversion recovery (FLAIR). Additionally, the patient received 7 T MRI (MAGNETOM, Siemens Healthcare, Erlangen, Germany) with a 1Tx/32Rx phased-array head coil and bilaterally placed dielectric pads. The acquired sequences were B1 mapping; T1w magnetization prepared 2 rapid gradient echo (MP2RAGE); FLAIR; T2-weighted; and resting-state blood-oxygen-level-dependent functional MRI (rs-fMRI). Furthermore, the images of 48 healthy controls (51.8 ± 11.4y, 26 females) were used with matching parameters for the T1w and rs-fMRI [12]. Sequence parameters can be found in Table 1.

**Table 1:** Sequence parameters. T1w: T1-weighted; rs-fMRI: resting-state functional MRI; TFL: turbo flash; TFE: turbo flash echo; TIR: turbo inversion recovery; MP2RAGE: magnetization prepared 2 rapid gradient echoes; TR: repetition time; TE: echo time; TI: inversion time; FOV: field of view; PE: phase encoding.

**Scanning parameters**		**B1 map**	**T1w**	**FLAIR**	**T2w**	**rs-fMRI**	**rs-fMRI (topup)**
3 T	Sequence	−	3D TFE	TIR	2D TSE	−	−
	**Voxel size [mm]**	−	1 x 1 x 1	0.43 x 0.43 x 0.6	0.45 x 0.45 x 5	−	−
	**Matrix size [FOVx x FOVy x slices]**	−	170 x 240 x 240	576 x 576 x 283	512 x 512 x 28	−	−
	**TR [ms]**	−	8.3	8000	2656	−	−
	**TE [ms]**	−	3.8	335	80	−	−
	**TI [ms]**	−	−	2400	−	−	−
	**Flip angle [deg]**	−	8	90	90	−	−
	**Phase enc. dir.**	−	A ≫ P	A ≫ P	L ≫ R	**−**	**−**
	**Scan orientation**	−	Transverse	Transverse	Transverse	−	−
	**Acceleration mode**	−	SENSE	SENSE	SENSE	−	−
	**Accel. factor PE**	−	3	2.2	3	−	−
	**Accel. factor 3D**	−	1	1	1	−	−
	**Multi-band acc. fac.**	−	−	−	−	−	−
	**Number of measurements**	−	1	1	1	−	−
	**Acquisition time [min:sec]**	−	03:26	05:44	01:52	**−**	**−**
**7 T**	**Sequence**	TFL	3D MP2RAGE	3D SPACE	SPACE	2D MBEPI	2D MBEPI
	**Voxel size [mm]**	3.9 x 3.9 x 5.0	0.7 x 0.7 x 0.7	0.8 x 0.8 x 0.8	0.6 x 0.6 x 2	1.4 x 1.4 x 1.4	1.4 x 1.4 x 1.4
	**Matrix size [FOVx x FOVy x slices]**	64 x 64 x 36	320 x 320 x 240	320 x 320 x 208	320 x 320 x 104	142 x 142 x 80	142 x 142 x 80
	**TR [ms]**	10,000	5030	8000	4000	2000	2000
	**TE [ms]**	2.24	2.47	303	283	18.8	18.8
	**TI [ms]**	−	900 | 2750	2330	−	−	−
	**Flip angle [deg]**	8	5 | 3	variable	variable	80	80
	**Phase enc. dir.**	A ≫ P	A ≫ P	A ≫ P	A ≫ P	**A** ≫ **P**	**P** ≫ **A**
	**Scan orientation**	Transverse	Sagittal	Sagittal	Transverse	Transverse	Transverse
	**Acceleration mode**	−	GRAPPA	GRAPPA	GRAPPA	GRAPPA	GRAPPA
	**Accel. factor PE**	−	3	4	2	3	3
	**Accel. factor 3D**	−	1	1	2	−	−
	**Multi-band acc. fac.**	−	−	−	−	2	2
	**Number of measurements**	1	1	1	1	200	10
	**Acquisition time [min:sec]**	00:20	08:07	12:18	03:34	**07:29**	**00:59**

### 2.3 Image pre-processing

The 7 T MP2RAGE T1-weighted (T1w) images underwent a custom preprocessing pipeline to generate MPRAGE-like, including bias-correction, multiplication by the MP2RAGE longer inversion image, and removal of non-brain tissue (Fig. S1). The 3 T T1w images were skull-stripped and bias-corrected. The rs-fMRI was corrected for geometric distortions and motion-related artifacts. Finally, the time series were bandpass filtered at 0.01–0.1 Hz. To study the temporal fluctuations related to neuronal activity, the following established rs-fMRI metrics were utilized: regional homogeneity (ReHo), amplitude of low frequency fluctuations (ALFF), and fractional ALFF (fALFF). Pre-processing details and toolbox references can be found in Supporting Information.

### 2.4 Automated morphometric analysis

The pre-processed T1w images from controls were utilized as a normative database against the patient’s pre-processed T1w. Morphometric Analysis Program (MAP18) software toolbox was utilized for automated morphometric analysis [7]. MAP18 outputs three statistical z-scores maps (i.e. how many standard deviations (SD) is one voxel deviant from the control data) sensitive to cortical blurring and thickening for the studied patient, and a combined image (a fusion of the three maps) to maximize sensitivity [7]. The resulting MAP18 combined image was thresholded at a z-score of higher than 1.96SD. This produced a binary mask of significantly abnormal areas. The largest abnormal region within the resected area was taken as the FCD mask (checked by an epilepsy-specialized neuroradiologist) and used for further calculations.

### 2.5 Statistical image analysis

The pre-processed T1w, ReHo, ALFF, fALFF, MAP18 combined z-score map, and the resection mask were coregistered to a 0.7 mm symmetrical MNI template (details in Supporting Information). Coregistered patient images were compared voxel-by-voxel with the control database, yielding a z-score map for each metric, where significantly higher z values (|z|>1.96SD) would indicate abnormal activity. Median z-scores in both the resection and the MAP18-defined FCD mask were calculated from each rs-fMRI metric. All processing was done in Python v3.11. Linear regression was fitted via the statsmodels package and the OLS module. Externally studentized residuals were extracted using the OLSinfluence class. The patient’s p-value was calculated using the scipy.stats.t.cdf (i.e. cumulative distribution function of the Student’s T random variable) for the absolute value of the patient's residual, multiplied by two for a two-tailed test. Since this single observation has no variance, visual assessment is crucial. Significance threshold was p < 0.05.

### 2.6 Outlier handling

Cook’s distance was used to determine outliers in the rs-fMRI linear regression residuals amongst the controls [13]. The distances for each residual were calculated using the OLSinfluence class. The outlier threshold was set to 5 times the mean Cook’s distance of the control residuals. The resulting threshold 0.1 yielded one outlier with Cook’s distance 0.31 amongst the controls. This outlier was removed from the analysis, thus resulting in 47 healthy controls (52 ± 11.4y; 25 females).

## 3 Results

### 3.1 Patient surgical workup and follow-up

**Fig. 2:**
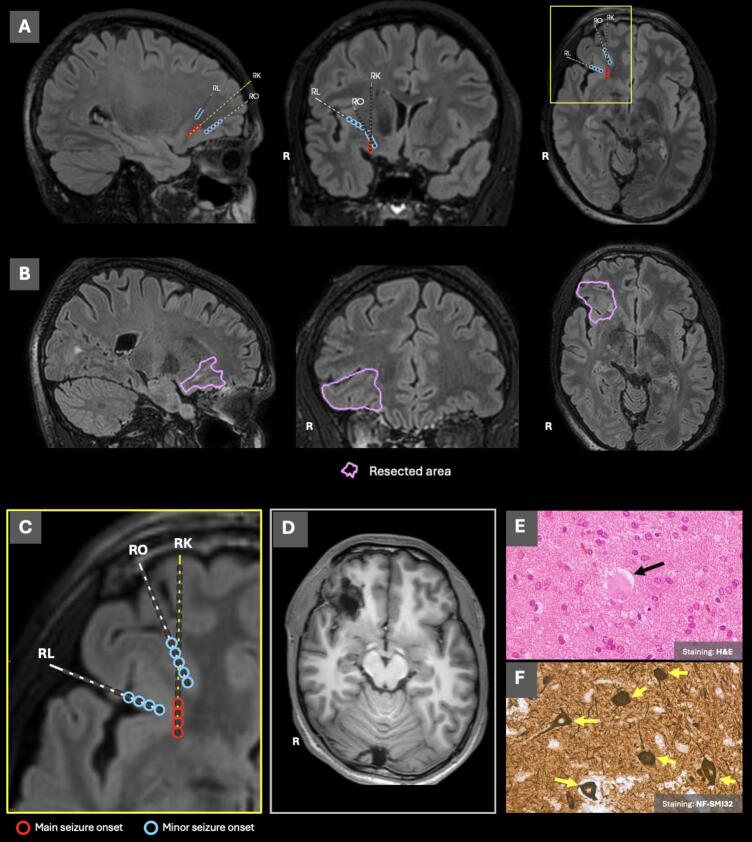
A: Visualization of the 3 T FLAIR with overlayed depth electrode positioning using MIND and DENSE (CNSprojects, Amsterdam) [14]. Only electrodes involved in the seizure onset are visualized, with the most prominent contacts highlighted – red circle indicates main seizure onset; light blue circle indicates minor seizure onset (i.e. not always involved directly at the seizure onset). B: delineation (purple contour) of the resected area overlaying the post-SEEG 3 T FLAIR. C: magnification (yellow box) of the electrode placement in transverse plane. The 3 T FLAIR sequence was used for both the electrode placement planning and the delineation of the resection area. D: post-resection 3 T FLAIR image. E,F: histological images of the resected specimen showing a balloon cell (E – black arrow) and dysmorphic neurons (F – yellow arrows). FLAIR: fluid-attenuated inversion recovery image. (For interpretation of the references to colour in this figure legend, the reader is referred to the web version of this article.)

Epilepsy onset occurred at age of 4 years with daily focal seizures with impaired awareness and secondary generalized tonic-clonic seizures. The patient, who was right-handed with normal NPE, underwent comprehensive preoperative evaluation with both 3 T and 7 T MRI being assessed negative (Fig. 1). MEG and SEEG findings indicated an EZ in the right frontobasal area and anterior insula. Radiofrequency thermal coagulation was performed on the depth-electrode contacts involved in the seizure onset (Fig. 2), after which the patient remained seizure-free for 3 months. Following informed consent, a tailored resection of the right frontobasal and anterior-insular region was performed in January 2024. Histopathology revealed FCD type IIb [5] (Fig. 2E, F). With currently 11 months follow-up after surgery, the patient is still seizure-free (ILAE score 1). The patient had an NPE prior to surgery however did not yet undergo the post-surgical re-evaluation. The patient did not report subjective changes in their cognitive functioning after the surgery.

### 3.2 Image post-processing

**Fig. 3:**
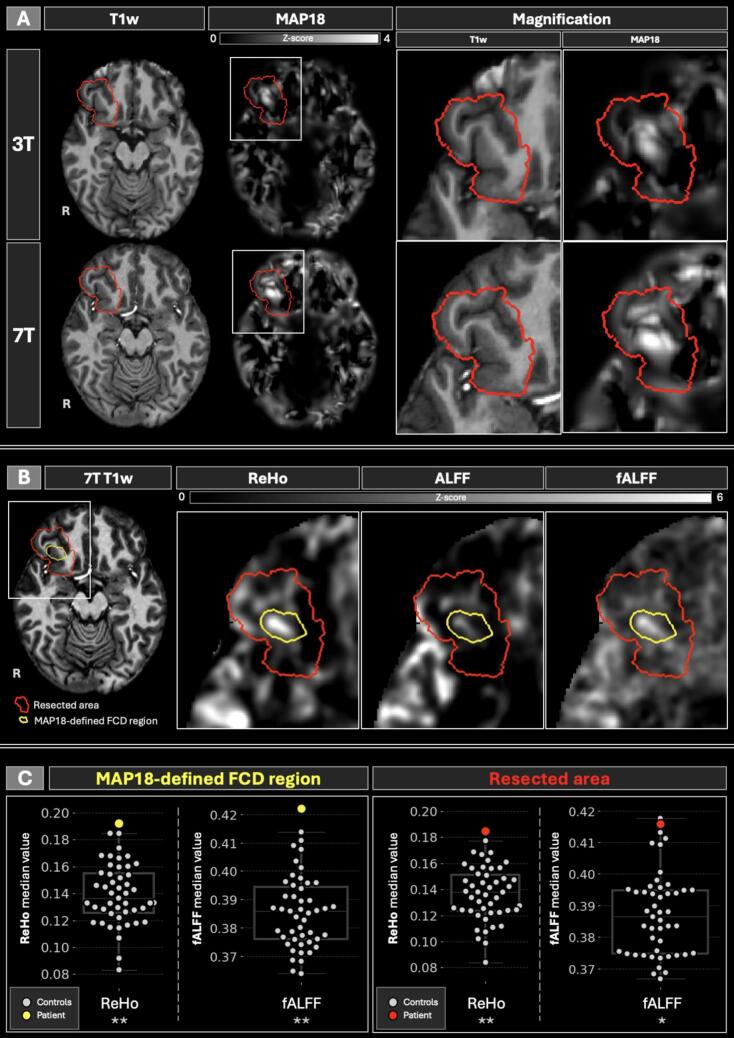
A: Morphometric MAP18 combined z-score maps for both 3 T and 7 T, with the delineation of the resected area (red contour). B: rs-fMRI z-score maps with the resected area (red contour) and the MAP18-defined FCD region (yellow contour) outlined. **C**: Comparison between the median values (plotted on y-axis) from the respective regions of interest, from only those metrics where the patient’s value was significantly abnormal with respect to the controls. * marks p < 0.05; ** marks p < 0.01. T1w: T1-weighted; MAP18: Morphometric Analysis Program; rs-fMRI: resting-state functional MRI; ReHo: regional homogeneity; ALFF: amplitude of low-frequency fluctuations; fALFF: fractional ALFF; FCD: focal cortical dysplasia. (For interpretation of the references to colour in this figure legend, the reader is referred to the web version of this article.)

MAP18 and rs-fMRI results are shown in Fig. 3. The largest MAP18 cluster encompassed 8.7 % of the resection mask and was overlaying the bottom of the right anterior insular sulcus (lateral orbitofrontal cortex; Fig. 1, Fig. 2). ReHo and fALFF z-scores were elevated in the MAP18-defined area. Note that multiple abnormalities were found outside the resection area. Quantitatively, ReHo and fALFF median values were found significantly higher compared with the controls both within the MAP18-defined FCD region (p = 0.004 and p = 0.005, respectively) and the entire resected area (p = 0.006 and p = 0.024, respectively) (Fig. 3).

## 4 Discussion

In this case illustration, we describe the added clinical value of 7 T structural and functional MRI with adequate image post-processing in a patient with MRI-negative DRE and a histopathologically-validated FCD type IIb. MAP18 applied to pre-processed T1w was found positive both at 3 T and 7 T, with higher conspicuity at 7 T. We also observed higher ReHo and fALFF values within the resected area and the MAP18-defined FCD region.

The patient underwent resective surgery of the right frontobasal and anterior insular regions in January 2024 after an extensive presurgical workup. After 11-month follow-up, the patient remains seizure free (ILAE score 1). At time of surgery, the patient was considered MRI-negative on both 3 T and 7 T. Initially, the 4-year-old MAP18 (prior to the 7 T MRI) was rated negative. A MAP18 finding requires an abnormality visible by neuroradiologists in structural scans to become “positive”. At that time, the available 3 T structural sequences did not provide enough supporting evidence. Furthermore, multidisciplinary epilepsy surgery conference has to confirm the EZ location, otherwise the finding will be considered false positive. Lastly, standard practice often skips T1w pre-processing before MAP18, though pre-processing may increase sensitivity. Here, we applied bias-field correction and brain extraction to the 3 T T1w before MAP18. This, along with the clinical assessment, is probably the reason why the original result was negative.

Upon visual inspection of our new MAP18 results, both 3 T and 7 T showed multiple abnormalities within the resected area with the largest cluster overlapping the presumed FCD location. The 7 T MAP18 results provided more detail and showed higher z-score values in the largest cluster overlaying the presumed EZ. Furthermore, the largest cluster corresponded with depth-electrode contacts involved in the seizure onset. Therefore, the largest cluster was taken as the FCD mask for image analysis and any additional clusters within the resected area were discarded.

Analysis of rs-fMRI was conducted in both the 7 T MAP18-defined FCD region and resected area, in both showing significantly increased (median) ReHo and fALFF values. The value of these metrics for lateralizing the EZ has been shown previously [15], however localization of FCDs remains uncertain. Previously, using 3 T MRI, Hong et al. found decreased ReHo and ALFF in FCD type IIb [16], which was supported by Jin et al. who found decreased ALFF and ReHo in perilesional grey matter [17]. The fALFF shows the relative power within a given frequency range, thus providing an indirect indicator of oxygen uptake and neuronal activity. We speculate that, as ReHo measures local synchronicity, increased neuronal synchronization within the EZ could indicate abnormal uptake of oxygen to facilitate the increased activity hinted towards by fALFF. This phenomenon is supported by previous findings where both ReHo and fALFF were found increased in glucose hypometabolic regions [18].

Regarding limitations, this study did not consider the time delay between the last seizure and the scanning session. It is well-known that seizures can induce transient structural and functional abnormalities which may impact the analysis [19]. While we do consider the MAP18 finding to be indicative of the FCD, the histopathological evaluation remains the ground truth for the presence of pathological tissue within the resected area. Single reviewer of the 7 T MRI observed a co-localized hyperintensity in the T2-weighted and cortical blurring in the 7 T FLAIR (Fig. 1). While these observations were deemed negative by the epilepsy surgery conference, our results support this initial finding. As this is a single case study, broad conclusions about rs-fMRI's utility for FCD localization cannot be drawn, though ReHo and fALFF show clear co-localized abnormality with 7 T MAP18.

Furthermore, the increased visibility of abnormalities observed in the retrospective MAP18 analysis at 7 T versus 3 T may be influenced by the differences in T1w acquisition voxel size. While the MP2RAGE at 7 T used 0.7 mm cubic voxels, the 3 T MPRAGE was acquired at 1.0 mm cubic. A direct comparison study using matched voxel sizes at both 3 T and 7 T would be valuable for future research, as both resolutions are achievable at both field strengths. Previously, it has been shown that MPRAGE and MP2RAGE demonstrate different sensitivities in FCD detection rate at 3 T [20]. To address this, we implemented the MP2RAGE pre-processing pipeline with the goal of creating a more MPRAGE-like contrast.

Although there are decades of development behind 3 T MRI in the presurgical workup, the ongoing implementations of 7 T MRI may also lead to reconsidering and improving the current (pre-)clinical workflows at 3 T MRI, for instance including a MP2RAGE scan. Currently, the availability of 7 T scanners is significantly lower compared with 3 T scanners which complicates establishing generalizable image processing pipelines and clinical workflows. Additionally, the associated examination costs are higher at 7 T compared with 3 T. The diagnostic efficacy is an actual topic of clinical research [9]. It is obvious that adequate image processing helps to improve the visibility of epilepsy related lesions both at 3 T and 7 T. However, 7 T has a clear advantage for the better spatial resolution and stronger magnetic susceptibility effects than 3 T, which cannot be compensated by image processing of 3 T images. Future studies need to demonstrate that subtle lesions can be detected or delineated more easily at 7 T than 3 T.

In case both MAP18 and rs-fMRI are available, especially in MRI-negative cases with limited prior knowledge on the EZ localization, the large number of abnormalities needs to be considered, especially regarding the rs-fMRI metrics. While the 7 T rs-fMRI shows promising results in the presented patient, similar results might have also been observed for 3 T rs fMRI if it would be part of clinical scanning protocol. Nevertheless, the results suggest rs-fMRI metrics confirm the MAP18 abnormality, though independent focus localization through only rs-fMRI remains challenging. Currently, no unified methodology exists for utilizing rs-fMRI metrics in the presurgical workup without prior knowledge of the EZ location. The numerous abnormalities outside the resected region make it difficult to identify a single focal seizure onset zone with abnormal BOLD activity. This emphasizes the need for multimodal presurgical workup and combining multiple diagnostic methodologies to filter out irrelevant signals. While the current case study clearly hints towards the potential utility of UHF rs-fMRI, its value for localizing FCDs across patients still needs to be further established.

## 5 Conclusion

The value of UHF MRI and advanced image post-processing was demonstrated in a case with originally 3 T and 7 T MRI-negative DRE who remained seizure-free 11 months post-surgery. FCD IIb was confirmed through histology. Retrospective evaluation revealed structural and functional abnormalities within the resected area. MAP18 after pre-processing of the T1w was positive on both 3 T and 7 T, with better visual conspicuity at 7 T. The rs-fMRI metrics, especially ReHo and fALFF, showed co-localized abnormalities with MAP18. This warrants further research into UHF MRI post-processing and rs-fMRI metrics in presurgical workup. Extended cohort studies could establish the added value of rs-fMRI in order to confirm subtle epileptogenic lesions, potentially improving postsurgical seizure freedom and patient quality of life.

## Ethical statement

We hereby confirm that:

1) the work described has not been published previously
2) the article is not under consideration for publication elsewhere
3) the article's publication is approved by all authors and tacitly or explicitly by the responsible authorities where the work was carried out
4) if accepted, the article will not be published elsewhere in the same form, in English or in any other language, including electronically, without the written consent of the copyright-holder

## Declaration of Generative AI and AI-assisted technologies in the writing process

During the preparation of this work the authors used Claude (https://claude.ai) in order to spell-check the text, improve conciseness, and facilitate better reading flow. After using this tool/service, the authors reviewed and edited the content as needed and take full responsibility for the content of the published article.

## CRediT authorship contribution statement

**Daniel Uher:** Writing – review & editing, Writing – original draft, Visualization, Software, Resources, Methodology, Investigation, Formal analysis, Data curation, Conceptualization. **Gerhard S. Drenthen:** Writing – review & editing, Visualization, Validation, Supervision, Project administration, Methodology, Investigation, Formal analysis, Data curation, Conceptualization. **Christianne M. Hoeberigs:** Writing – review & editing, Visualization, Validation, Methodology, Investigation, Formal analysis, Data curation. **Rick H.G.J. van Lanen:** Writing – review & editing, Validation, Resources, Methodology, Investigation, Funding acquisition, Data curation. **Albert J. Colon:** Writing – review & editing, Visualization, Validation, Supervision, Resources, Project administration, Methodology, Investigation, Funding acquisition, Formal analysis, Data curation, Conceptualization. **Roy A.M. Haast:** Writing – review & editing, Visualization, Methodology, Data curation. **Vivianne H.J.M. van Kranen-Mastenbroek:** Writing – review & editing, Visualization, Investigation, Formal analysis, Data curation. **Guido Widman:** Writing – review & editing, Writing – original draft, Visualization, Software, Methodology, Investigation, Formal analysis, Data curation. **Paul A.M. Hofman:** Writing – review & editing, Visualization, Validation, Resources, Methodology, Investigation, Formal analysis, Data curation. **Louis G. Wagner:** Writing – review & editing, Validation, Resources, Investigation, Formal analysis, Data curation. **Jan C. Beckervordersandforth:** Writing – review & editing, Visualization, Validation, Investigation, Formal analysis, Data curation. **Jacobus F.A. Jansen:** Writing – review & editing, Visualization, Validation, Supervision, Resources, Project administration, Methodology, Investigation, Funding acquisition, Formal analysis, Data curation, Conceptualization. **Olaf E.M.G. Schijns:** Writing – review & editing, Visualization, Validation, Supervision, Resources, Project administration, Methodology, Investigation, Funding acquisition, Formal analysis, Data curation, Conceptualization. **Walter H. Backes:** Writing – review & editing, Visualization, Validation, Supervision, Resources, Project administration, Methodology, Investigation, Funding acquisition, Formal analysis, Data curation, Conceptualization.

## Funding

This work was supported by the Dutch epilepsy foundation (*EpilepsieNL*) with grant number [WAR project number 2020-09].

## Declaration of competing interest

The authors declare that they have no known competing financial interests or personal relationships that could have appeared to influence the work reported in this paper.

## Appendix

Members of the Academic Center for Epileptology (ACE) study group:

• Gwendolyn de Bruyn (child neurologist),
• Albert Colon (neurologist/clinical neurophysiologist),
• Jim Dings (neurosurgeon),
• Marc Hendriks (Clinical neuropsychologist),
• Danny Hilkman (neurologist),
• Christianne Hoeberigs (radiologist),
• Jochem van der Pol (nuclear medicine physician),
• Lotte de Jong (Physician assistant),
• Kim Rijkers (neurosurgeon),
• Sylvia Klinkenberg (child neurologist),
• Vivianne van Kranen Mastenbroek (neurologist/clinical neurophysiologist),
• Jeske Nelissen (nurse practitioner AZG),
• Pieter Kubben (neurosurgeon),
• Walter M. Palm (neuroradiologist),
• Rob P.W. Rouhl (neurologist),
• Olaf Schijns (neurosurgeon),
• Simon Tousseyn (neurologist),
• Marielle Vlooswijk (neurologist),
• Louis Wagner (neurologist),
• Dorien Weckhuysen (neurologist),
• Ilse van Straaten (clinical neurophysiologist).
