# Supplementary material for: The role of ultra-high field MRI and image processing in the presurgical workup in MRI-negative focal epilepsy: A validated 7T MRI case study
Source: Epilepsy Behav Rep. 2025 Mar 15;30:100761. doi: 10.1016/j.ebr.2025.100761 (PMC11964652; doi:10.1016/j.ebr.2025.100761)
Supplement: Supplementary Data 1 [file mmc1.docx]

**Supporting information**

*2.3 Image pre-processing – EXTENDED description*

For a given subject, the 7T MP2RAGE unified image (i.e. T1w) was bias-corrected using the acquired B1 map via the scripts by Marques and Zwiers [1]. Additionally, the MP2RAGE longer inversion image (i.e. proton density image) was further bias-corrected using SPM12 [2]. The facial features and dielectric pads were stripped from both the T1w and INV2 images using the FaceOff toolbox [3]. The T1w image was then multiplied by the bias-corrected INV2 image to generate more MPRAGE-like contrast. Finally, the MPRAGE-like T1w was stripped of non-brain tissue using FSL’s brain extraction tool (BET) [4]. The pipeline is visualized in Figure S1. The 3T T1w images were skull-stripped using FSL’s BET and bias-corrected using SPM12.

The rs-fMRI was corrected for geometric distortions using FSL’s topup correction [5,6]. Motion parameters were estimated using FSL’s mcflirt [6] and corrected using ICA-AROMA toolbox [7]. Finally, the time series were bandpass filtered at 0.01-0.1Hz using the RESTplus toolbox [8]. To study the temporal fluctuations related to neuronal activity, the following established rs-fMRI metrics were derived using the RESTplus toolbox [8]: regional homogeneity (ReHo), amplitude of low frequency fluctuations (ALFF), and fractional ALFF (fALFF).


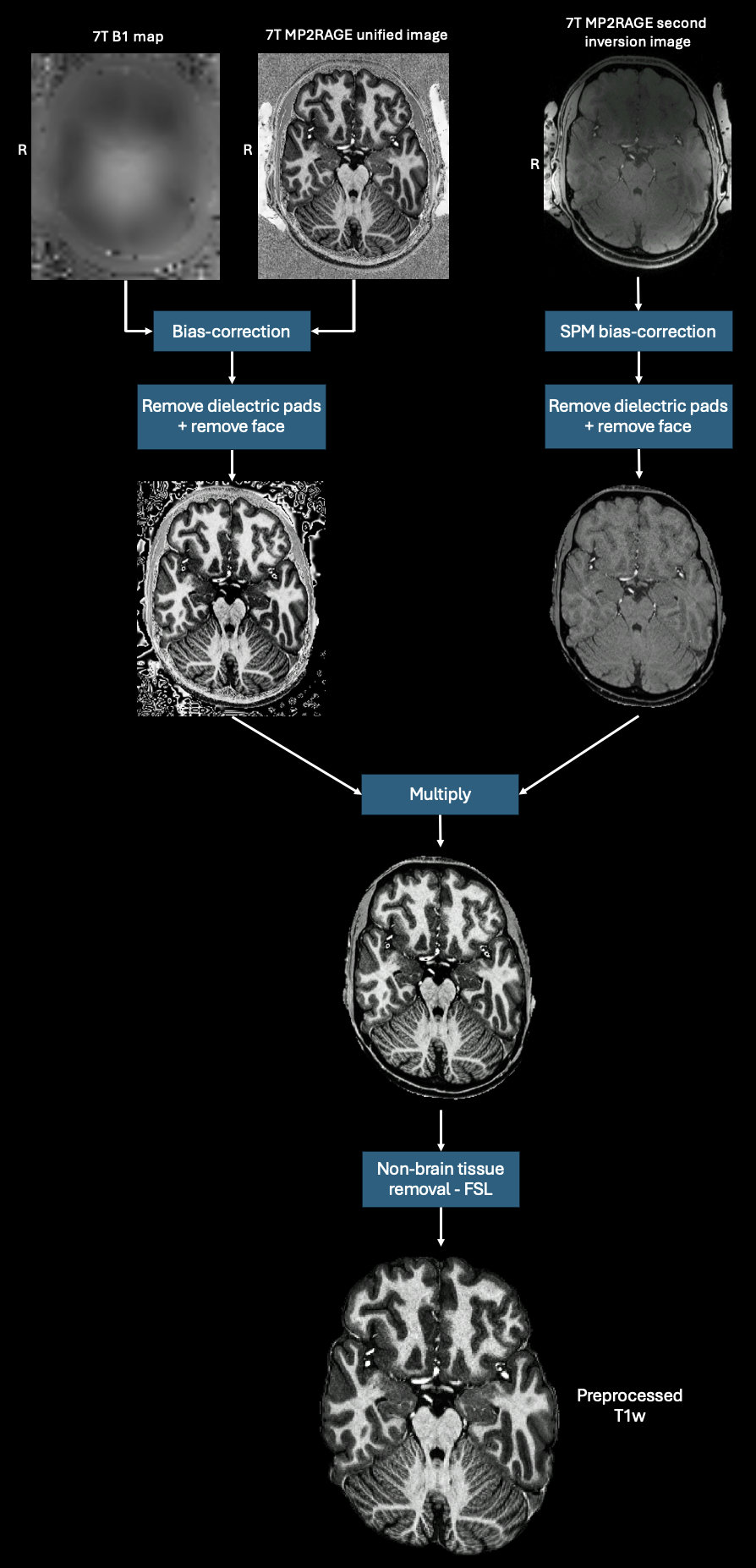


**Figure S1**: Preprocessing pipeline for the 7T T1-weighted images.

*Image coregistration*

The pre-processed T1w, ReHo, ALFF, fALFF, MAP18 combined z-score map, and the resection mask were coregistered to a 0.7mm symmetrical MNI template. The steps were:

1) *mri_vol2vol* [9] to move the functional metrics to the T1w space via the default qform/sform (i.e. the --regheader flag). This step was done only for those images that were not already in the T1w space (i.e. ReHo, ALFF, fALFF, resection mask);

2) two-fold *mri_coreg* [9], first with 6 degrees of freedom followed by 12 degrees of freedom;

3) *antsRegistrationSyNQuick.sh* [10] for the final non-linear warp to the MNI template. For ReHo, ALFF, fALFF, trilinear interpolation was used, while nearest neighbor was used for the resection mask.

**References**

[1] Marques JP, Zwiers M. MP2RAGE-related-scripts 2023. https://doi.org/10.5281/ZENODO.7845916.

[2] Friston KJ, editor. Statistical parametric mapping: the analysis of funtional brain images. 1st ed. Amsterdam ; Boston: Elsevier/Academic Press; 2007.

[3] Sriranga Kashyap. FaceOff 2023. https://doi.org/10.5281/ZENODO.8120900.

[4] Smith SM. Fast robust automated brain extraction. Human Brain Mapping 2002;17:143–55. https://doi.org/10.1002/hbm.10062.

[5] Smith SM, Jenkinson M, Woolrich MW, Beckmann CF, Behrens TEJ, Johansen-Berg H, et al. Advances in functional and structural MR image analysis and implementation as FSL. NeuroImage 2004;23:S208–19. https://doi.org/10.1016/j.neuroimage.2004.07.051.

[6] Jenkinson M, Beckmann CF, Behrens TEJ, Woolrich MW, Smith SM. FSL. NeuroImage 2012;62:782–90. https://doi.org/10.1016/j.neuroimage.2011.09.015.

[7] Pruim RHR, Mennes M, Van Rooij D, Llera A, Buitelaar JK, Beckmann CF. ICA-AROMA: A robust ICA-based strategy for removing motion artifacts from fMRI data. NeuroImage 2015;112:267–77. https://doi.org/10.1016/j.neuroimage.2015.02.064.

[8] Jia X-Z, Wang J, Sun H-Y, Zhang H, Liao W, Wang Z, et al. RESTplus: an improved toolkit for resting-state functional magnetic resonance imaging data processing. Science Bulletin 2019;64:953–4. https://doi.org/10.1016/j.scib.2019.05.008.

[9] Fischl B. FreeSurfer. NeuroImage 2012;62:774–81. https://doi.org/10.1016/j.neuroimage.2012.01.021.

[10] Avants, Brian B., Tustison N, Johnson HJ. Advanced Normalization Tools (ANTs) 2020. https://doi.org/10.5281/ZENODO.5138159.
